# High anxiety and depressive symptoms in partners of type 1 diabetes persons in a sample of the Brazilian population

**DOI:** 10.1186/s13098-020-00531-5

**Published:** 2020-03-24

**Authors:** E. Buin, E. J. Pavin, M. S. V. M. Silveira

**Affiliations:** 1 Internal Medicine Postgraduate Program, Faculty of Medical Sciences, State University of Campinas, Campinas, São Paulo, Brazil; 2 Endocrinology Division, Department of Internal Medicine, Faculty of Medical Sciences, State University of Campinas, Campinas, São Paulo, Brazil

**Keywords:** Diabetes Mellitus, Type 1, Partners, Anxiety, Depression

## Abstract

**Background:**

Type 1 diabetes (T1D) affects psychologically not only the persons with diabetes themselves but affects their family members. Few studies were conducted to investigate mental health in T1D partners. This study aims: (1) to investigate the frequency of depressive and anxiety symptoms in T1D partners and, (2) to investigate the associations among partners’ depressive and anxiety symptoms and their sociodemographic and behavioral characteristics, and (3) to investigate the associations among partners’ depressive and anxiety symptoms and clinical, laboratory and demographic characteristics of their T1D spouses in a Brazilian population.

**Methods:**

In a transversal study 72 T1D partners were interviewed. Partners were invited to take part in the study during their T1D spouses’ routine consultations. Those who consented to take part in the study signed the consent form. This study followed the principles of the Declaration of Helsinki and was approved by the University Ethics in Research Committee. Inclusion criteria were T1D partners age ≥ 18 and T1D diagnosis > 6 months. Exclusion criteria were cognitive impairment, history of major psychiatric disorders, and severe chronic and terminal diseases. Depressive symptoms were evaluated by the depression subscale of the Hospital Anxiety and Depression scale (HADD) and anxiety symptoms were evaluated by the anxiety subscale of the same instrument (HADA). T1D partners were divided into subgroups according to score ≥ 8 and < 8 in both subscales. Demographic and clinical data were obtained from interview. Descriptive analyses were undertaken using means and percentages, as appropriate. Differences between groups were assessed by the Mann–Whitney test for numerical variables, by the Chi Square test or by Fisher’s exact test for categorical variables, as appropriate. All analyses were undertaken using SAS version 9.2 for Windows. Statistical significance was set at 0.05.

**Results:**

Of all 72 T1D partners, 72.2% were male, mean age was 42.7 ± 14.1 years old, years of school attendance were 11.8 ± 3.9 years, and 48.5% had income reaching until 3 Brazilian minimal wages. Forty-three percent reported high anxiety symptoms (HADA ≥ 8) and 18.1% reported high depressive symptoms (HADD ≥ 8). Comparing T1D partners group with HADA ≥ 8 and < 8, the first one was associated with CGM use (41.94% vs 19.51%; p = 0.03). Similarly, comparing T1D partners group with HADD ≥ 8 and < 8, the first one was associated with (1) longer duration of T1D of their spouses (28.6 ± 7.1 vs 22.4 ± 12.2; p = 0.02); (2) less years of school attendance of T1D partners (9.3 ± 3.2 vs 12.3 ± 3.8; p = 0.02), and (3) higher number of hypoglycemic episodes requiring other person’s intervention (6.3 ± 8.9 vs 2.4 ± 4.7; p = 0.009). Seventy-six percent of partners who helped personally in their spouses’ hypoglycemia recovery had HADD ≥ 8 vs 44.7% with HADD < 8 (p = 0.03). Likewise, 84.6% vs 54.2% of partners in which their spouses have T1D chronic complications had HADD ≥ 8 and < 8, respectively (p = 0.04).

**Conclusion:**

This study showed a high frequency of relevant anxiety and depressive symptoms in this T1D partner population. Several issues related to T1D of their spouses were associated with these symptoms. These results emphasize the need to incorporate the psychological and psychiatric aspects into T1D partners’ education and care.

## Background

Type 1 Diabetes (T1D) is a chronic disease that requires a daily self-care and the adoption of specific behaviors to appropriately manage the disease: it demands frequent glycemic monitoring, insulin dose adjustment and vigilance due to risks of hypoglycemia and hyperglycemia [1]. The complexity of T1D management, all the tasks involved in glycemic control and the possibility of the appearance of various clinic complications are important sources of distress and psychological suffering in T1D persons [2]. The diabetes-related distress also called diabetes distress (DD) is present in some level in almost everyone living with T1D [2] and it may interfere in patient’s ability of T1D self-management [2–6]. Many T1D individuals mention the frustration with the onus to manage their disease and they experience concerns, anger, fears, and feelings of “diabetes burnout’’ [7]. Moreover, the frequency of anxiety symptoms, depressive symptoms and depression are higher in T1D patients [3, 8–10].

Despite being well described in the literature that specific behaviors and lifestyle changes required for diabetes self-management not only affects T1D patients themselves but also affects their family members, few studies have evaluated the burden of the disease to family members. The required tasks to keep the glycemic targets may conflict with the pre-established family routine [11]. The need for developing new eating habits, absence from work to accompany family members during medical appointments, redefining family finances, and many other changes can be stressful [12].

Most of the researches with a focus on family evaluated the psychological impact on parents of teenagers or children with diabetes and the effect of family support on the disease prognostic [13]. Few studies investigated the mental health of family members of adults with diabetes [13]. In addition, in the adult population with diabetes, studies were focused on the effects regarding family support related to diabetes outcomes [14–16], and not in the impact of diabetes in mental health of family members. Studies conducted with type 2 diabetes patients (T2D) pointed out that diabetes brings a psychological impact for family, causing much concern, high level of suffering and reduction of emotional well-being [17, 18].

Fisher et al. [2] showed that there is a high level of emotional suffering for partners of patients with diabetes, so high or even higher than for the own patients, mainly, if the partner is female gender. The Diabetes Attitudes, Wishes and Needs 2 study (Dawn 2), which includes T1D and T2D patients emphasized that the psychosocial impact of diabetes over family members and the psychosocial problems of family members were obstacles for their effective involvement in diabetes self-management [19, 20]. Moreover, this study highlighted that the actual health systems are not well-equipped to offer support or educate families of persons with diabetes [19].

Research exclusively with T1D patients and their partners demonstrated that T1D affects psychologically not only persons with diabetes but also their partners or spouses [7]. Even though partners’ involvement are variable, high levels of anxiety, especially related to hypoglycemia and fears of future complications was described. It brings exhaustion to partners themselves as well to their relationships [1].

Gonder-Frederick et al. [21] highlighted that T1D partners demonstrated fear related to hypoglycemia in a higher average than the own patients. Severe cases of recent hypoglycemia episodes in their spouses brought a significant greater fear of new episodes, marital conflict related to diabetes treatment and sleep disturbance due to concerns about risks of hypoglycemia [21]. Van Dijk et al. [22] showed that the presence of nocturnal hypoglycemias may compromise the quality of sleep of T1D persons, and, consequently, it can affect sleep of their partners [23]. The disturbance of sleep can negatively affect daily functional related to emotional, cognitive performance, and alert behavior, bringing vulnerability to deal with daily stressors.

Therefore, the knowledge of the sources of psychological suffering in this population can direct more appropriate psychological interventions for them. The goals of this study were: (1) to investigate the frequency of depressive and anxiety symptoms in T1D partners, (2) to investigate the associations among partners’ depressive and anxiety symptoms and their sociodemographic and behavioral characteristics, and 3-to investigate the associations among partners’ depressive and anxiety symptoms and clinical, laboratorial and demographic characteristics of their T1D spouses in a Brazilian population.

## Methods

In a cross-sectional study, we evaluated partners of T1D patients receiving outpatient care at University of Campinas Tertiary Hospital Diabetes Clinic and in a Private Diabetes Clinic in Campinas, Brazil. T1D partners were interviewed between March 2018 and March 2019 and they were invited to participate in this study during medical appointments of their T1D spouses. Those who consented to participate signed the consent form. This study followed the principles of the declaration of Helsinki and it was approved by the Ethics and Research Committee of the University in May 2017 (CAAE number: 68202017.0.0000.5404).

Inclusion criteria were: T1D partners with age 18 years old and older and T1D diagnosis of their spouses for at least 6 months. Exclusion criteria were: a cognitive impairment that could affect the partners’ ability to answer the protocol questions, partners with a history of major psychiatric disorders (such as schizophrenia, drug addiction, dementia) and with serious chronic diseases, which cause them high psychologic impact.

Depressive symptoms were evaluated by the depressive subscale of Hospital Anxiety and Depression Scale (HAD-D) and anxiety symptoms were evaluated by the anxiety subscale of the same instrument (HAD-A). This instrument was created by Zigmond et al. [24] and it was translated and validated into Portuguese by Botega et al. [25]. Each subscale has 7 items and each one is graded from 0 to 3. Bjelland et al. [26] through a systematic review of literature, identified a point of cut of 8, to indicate the presence of clinically relevant depressive and anxiety symptoms.

T1D partners were divided into subgroups according to score ≥ 8 e < 8 in both subscales. Partners’ behavioral and sociodemographic data and T1D patients’ clinical and sociodemographic data were collected through structured interviews (questionnaire developed for this study). T1D partners data were age (years), gender, years of scholarity, income based on wage range according to Brazilian Institute Geography and Statistics (IBGE) [27]. The behavioral aspect of partners was the fact that they helped their spouses recover from severe episodes of hypoglycemia. Only objective data, more specifically, clinical and socio-demographic aspects were collected of the T1D patients through the partners’ reports. The data included: age (years), gender, years of scholarity, years of T1D, continuous glucose monitoring (CGM) use, Continuous Insulin Infusion pump use, presence of chronic complications (nephropathy, retinopathy, peripheral neuropathy), occurrence of ketoacidosis since the diagnosis of the disease, and number of severe episodes of hypoglycemia in the last 6 months. Diabetic retinopathy was diagnosed based on fundoscopy examinations performed by the University Ophthalmology Department. Nephropathy was diagnosed if two or more urine samples separated by at least 30 days showed “albumin/creatinine ratio above 30 mg/g”. Neuropathy was diagnosed based on annual clinical examinations performed by the staff physicians at the diabetes clinic. The hypoglycemia episodes were classified in severe and not severe, based on the need of receiving help from others or not [28–30].

T1D patients laboratorial data were collected based on chart review. Glycemic control was expressed by glycated hemoglobin (HbA1c)—HPLC method (High Performance Liquid Chromatography.

### Statistical methods

Descriptive analyses were performed with measures of means and medians for numerical variables and frequency (percentage) for categorical variables. Differences between groups were assessed by the Mann–Whitney test for numerical variables, by the Chi Square test or by Fisher’s exact for categorical variables, as appropriate. All analyses were undertaken using SAS version 9.4 for Windows. Statistical significance was set at 0.05.

## Results

Of all 72 T1D partners recruited, 69.4% were male gender, mean age was 42.69 ± 14.1 years old, mean education level was 11.8 ± 3,9 years, and 45.83% had income reaching until 3 minimum Brazilian wages. Moreover, of all 72 T1D patients, 72.2% were female gender, mean age was 41.18 ± 12.74 years old, and mean of T1D duration was 23.53 ± 11.69 years. Table 1 shows the sociodemographic characteristics and behavioral factors of T1D partners while the sociodemographic, clinical and laboratorial aspects of T1D patients were summarized in Table 2.

**Table 1:** Sociodemographic characteristics and behavioral factors of T1D partners

Total N = 72				
Variables	N (%)	Mean	Median	SD
Age (years)	–	42.69	40.00	14.09
Male gender	50 (69.4%)	–	–	–
Education level (years of study)	–	11.81	11.00	3.91
Income reaching until 3 Brazilian minimum wages	33 (45.8%)			
Partner helped T1D spouse recover from severe episodes of hypoglycemia	36 (50.0%)	–	–	–
Length of relationship (years)	–	14.74	12.00	12.41

*T1D* type 1 diabetes, *SD* standard deviation; severe episodes of hypoglycemia: episodes of hypoglycemia that T1D patients needed assistance from others to recover

**Table 2:** Sociodemographic, clinical and laboratory characteristics of T1D patients

Total N = 72				
Variables	N (%)	Mean	Median	SD
Age (years)	–	41.18	39.00	12.74
Female gender	52 (72.22)	–	–	–
Education level (years of study)	–	11.68	11.00	4.05
Years of T1D	–	23.53	23.00	11.69
HbA1c (%)	–	8.19	8.00	1.53
CGM use	21 (29.17)	–	–	–
Insulin pump use	12 (16.77)	–	–	–
Multiple insulin doses	60 (83.33)	–	–	–
Presence of chronic complications	43 (53.72)	–	–	–
Occurrence of ketoacidosis since the T1D diagnosis of the disease	26 (36.11)	–	–	–
Number of severe episodes of hypoglycemia (last 6 months)	–	3.14	1.00	5.82

*T1D* type 1 diabetes, *SD* standard deviation, *HbA1c* glycated hemoglobin, *CGM* continuous glucose monitoring; Chronic Complications (0–3): nephropathy, retinopathy, peripheral neuropathy; severe episodes of hypoglycemia: episodes of hypoglycemia that T1D patients needed assistance from others to recover

### Frequency of T1D partners clinically relevant anxiety and depressive symptoms

**Fig. 1:**
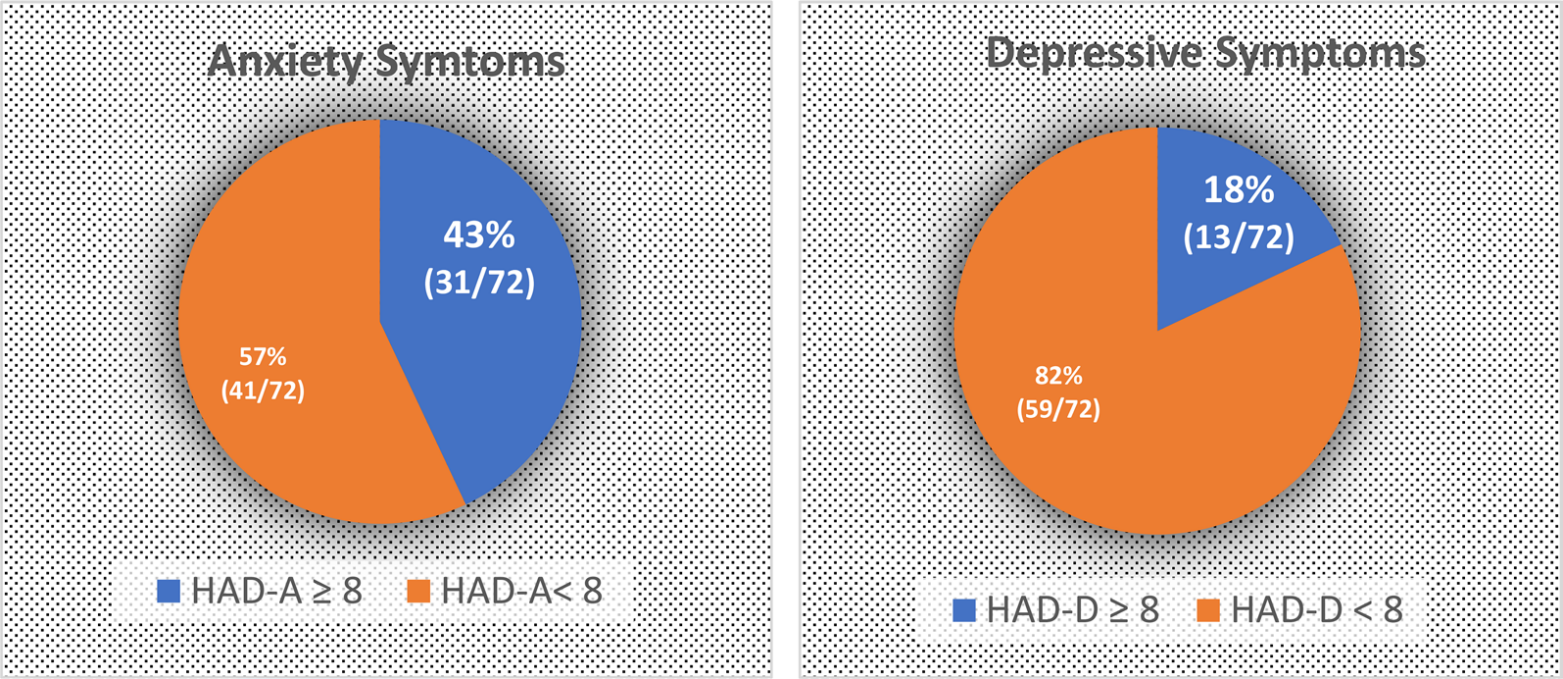
Frequency of T1D partners clinical relevant anxiety and depressive symptoms

High anxiety symptoms (HAD-A ≥ 8) were observed in 43% of T1D partners and 18.1% of T1D partners had high depressive symptoms (HADD ≥ 8), indicating high anxiety and depressive levels in this T1D partners population (Fig. 1).

### Associations among T1D partners anxiety and depressive symptoms and variables

Comparing T1D partners groups with HADA ≥ 8 e < 8, the first one was associated to use of CGM (41.94% vs 19.51%; p = 0.0382). No differences were found among other variables in this study in both groups. Mean partners’ age was 45.65 ± 15.38 vs 40.46 ± 12.77, p = 0.1135; mean partners’ education was 11.13 ± 4.23 vs 12.33 ± 3.62, p = 0.0548; length of relationship was 16.81 ± 14.13 vs 13.17 ± 10.84; p = 0.3964; Partners’ gender male 67.74% vs 70.73%, p = 0.7851; income reaching until 3 Brazilian minimal wages 38.71% vs 51.22%, p = 0.2915.

Partners who helped their T1D spouses recover from severe episodes of hypoglycemia in the last 6 months 61.29 vs 41.46, p = 0.0957.

The sociodemographic, clinical and laboratorial characteristics of T1D patients and partners in both groups are summarized in Table 3.

**Table 3:** Descriptive data analysis comparing partners with scores < and ≥ 8 in HAD-A subscale (anxiety symptoms)

T1D partners/N = 72	Partners HAD-A < 8 N = 41/72	Partners HAD-A ≥ 8 N = 31/72	
Variables	Mean ± SD	Mean ± SD	p-valor
Age (years)	40.46 ± 12.77	45.65 ± 15.38	0.1135^a^
Education level (years of study)	12.33 ± 3.62	11.13 ± 4.23	0.0548^a^
Length of relationship (years)	13.17 ± 10.84	16.81 ± 14.13	0.3964^a^

Variables	N (%)	N (%)	p-valor
Gender male	29 (70.73)	21 (67.74)	0.7851^b^
Income reaching until 3 Brazilian minimal wages	21 (51.22)	12 (38.71)	0.2915^b^
Helped T1D spouses recover from severe episodes of hypoglycemia in the last 6 months	17 (41.46)	19 (61.29)	0.0957^b^

T1D patients/ N = 72			
Variables	Mean ± SD	Mean ± SD	p-valor
Age (years)	39.22 ± 13.03	43.77 ± 12.07	0.1330^a^
Education level (years of study)	12.32 ± 4.14	10.84 ± 3.84	0.1633^a^
Years of T1D	23.93 ± 11.49	23.00 ± 12.12	0.7630^a^
Number of severe episodes of hypoglycemia in the last 6 months	2.39 ± 4.65	4.13 ± 7.05	0.1193^a^
HbA1c (%), N = 71	8.24 ± 1.62	8.16 ± 1.50	0.9814^a^

Variables	N (%)	N (%)	p-valor
Female gender	30 (73.17)	22 (70.97)	0.8363^b^
Multiple insulin doses	32 (78.05)	28 (90.32)	0.1664^b^
Insulin pump use	9 (21.95)	3 (9.68)	0.1664^b^
CGM use	8 (19.51)	13 (41.94)	*0.0382*^*b*^
Presence of chronic complications	21 (51.22)	22 (70.97)	0.0907^b^
Occurrence of ketoacidosis since the T1D diagnosis of the disease	13 (31.71)	13 (41.74)	0.3710^b^

Italic value indicates significance of p value (p < 0.05)

*HAD-A* Anxiety subscale of Hospital Depression and Anxiety Scale, *T1D* type 1 diabetes, *SD* standard deviation; severe episodes of hypoglycemia: episodes that the patient with type 1 diabetes needed assistance from others to recover, *HbA1c* glycated hemoglobin, *CGM* continuous glucose monitoring; chronic complications (0–3): nephropathy, retinopathy, peripheral neuropathy

^a^Mann–Whitney test, ^b^Chi-square test

Regarding depressive symptoms, comparing T1D partners groups with HAD-D ≥ 8 e < 8, the first one was associated with: (1) T1D duration of their spouses (28.62 ± 7.11 vs 22.41 ± 12.24; p = 0.0265); (2) T1D partners with lower education level (9.38 ± 3.28 vs 12.35 ± 3.86; p = 0.0206); and (3) greater number of hypoglycemia episodes which required others persons intervention to recover (6.38 ± 8.90 vs 2.42 ± 4.72; p = 0.0092). Seventy-six percent of T1D partners who personally helped in their spouse’s hypoglycemia recovery had HAD-D ≥ 8 vs 44% with HAD-D < 8 (p = 0.0320). Similarly, 84.6% vs 54.2% of T1D partners whose spouses had T1D chronic complications showed HAD-D ≥ 8 e < 8, respectively (p = 0.0432) (Table 4).

**Table 4:** Descriptive data analysis comparing partners with scores < and ≥ 8 in HAD-D subscale (depressive symptoms)

T1D partners/N = 72	Partners HAD-D < 8 N = 59/72	Partners HAD-D ≥ 8 N = 13/72	
Variables	Mean ± SD	Mean ± SD	p-valor
Age (years)	42.20 ± 13.35	44.92 ±17.51	0.6869^a^
Education level (years of study)	12.35 ± 3.86	9.38 ± 3.28	*0.0206*^*a*^
Length of relationship (years)	14.55 ± 12.54	15.62 ± 12.22	0.6549^a^

Variables	N (%)	N (%)	p-valor
Male gender	41 (69.49)	9 (69.23)	1.0000^c^
Income reaching until 3 Brazilian minimal wages	27 (45.76)	6 (46.15)	0.9796^b^
Helped T1D spouses recover from severe episodes of hypoglycemia in the last 6 months	26 (44.07)	10 (76.92)	*0.0320*^*b*^

T1D patients/N = 72			
Variables	Mean ± SD	Mean ± SD	p-valor
Age (years)	40.56 ± 13.01	44.00 ± 11.47	0.3715^a^
Education level (years of study)	12 ± 4.05	10.23 ± 3.90	0.1865^a^
Years of T1D	22.41 ± 12.24	28.62 ± 7.11	*0.0265*^*a*^
Number of severe episodes of hypoglycemia last 6 months	2.42 ± 4.72	6.38 ± 8.90	*0.0092*^a^
HbA1c, N=71	8.24 ± 1.62	7.97 ± 1.04	0.8700^a^

Variables	N (%)	N (%)	N (%)
Female gender	43 (72.88)	9 (69.23)	0.7457^c^
Multiple insulin doses	47 (79.66)	13 (100.00)	0.1071^c^
Insulin pump use	12 (20.34)	0 (0)	0.1071^c^
CGM use	16 (27.12)	5 (38.46)	0.5035^c^
Presence of chronic complications	32 (54.24)	11 (84.62)	*0.0432*^*c*^
Occurrence of ketoacidosis since the T1D diagnosis of the disease	22 (37.29)	4 (30.77)	0.7579^c^

Italic values indicate significance of p values (p < 0.05)

*HAD-D* Depressive subscale of Hospital Depression and Anxiety Scale, *T1D* type 1 diabetes, *SD* standard deviation; severe episodes of hypoglycemia: episodes that the patient with type 1 diabetes needed assistance from others to recover, *HbA1c* glycated hemoglobin, *CGM* continuous glucose monitoring; chronic complications: nephropathy, retinopathy, peripheral neuropathy

^a^Mann–Whitney test, ^b^Chi-square test, ^c^Fisher’s exact test

## Discussion

The present study found a high frequency of clinically relevant anxiety (43%) and depressive symptoms (18.1%) in T1D partners. The levels of these symptoms are higher compared to anxiety and depressive levels in a Brazilian general population (9.3% and 5.8%, respectively) [31]. Several issues were related to these symptoms in this population. Polonsky et al. [7], Trief et al. [1] reported positive association among depression, anxiety, and distress and family members of diabetes patients, emphasizing that T1D not only psychologically affects T1D persons but also affects their partners or spouses [7].

Similar findings were described by Trief et al. [1], in a qualitative study with T1D adults and their partners. This study identified high anxiety and distress, especially related to hypoglycemia and fears about diabetes complications in the future.

This current study also found an association between high anxiety symptoms in T1D partners whose spouses used CGM. Partners reported that audible alarms of the device during hypoglycemia and hyperglycemia episodes arouse not only T1D patients but also arouse them. That situation caused sleep disturbance and may increase anxiety levels. This aspect was also highlighted in the study of Tracy et al. [23]. Thus, daily sleep quality may be critical for couples who deal with T1D. The literature has been shown that sleeplessness and sleep disturbance may cause a negative impact in physical and mental well-being of individuals [32, 33], bringing deficits to cognitive function, reduction in the ability of concentration, psychological stress, damage to physical health and increased sensitivity [34, 35]. Despite sensors such as CGM bring several benefits, sleep interruption remains common and it might be increased by these devices [36].

The current study also found positive association among T1D partners clinically relevant depressive symptoms and the higher duration of their spouses disease. In addition, partners with lower education had high depressive levels. The higher time of T1D exposition with the complex daily tasks required to manage the disease brings a larger exposition to stressful situations (for example: frequent blood glucose tests, calculation of insulin doses according to carbohydrate count in each meal, surveillance due to the risk of hypoglycemia) causing a greater emotional burnout for patients as well as to partners. Moreover, the higher time of T1D increases the risks of chronic complications, which may cause greater levels of depressive symptoms. The presence of T1D chronic complications may create a need of assistance from partners and besides that, it may awaken the real fear or the fantasy of losing the loved one, increasing the risks of symptoms of the depressive sphere.

The association of partners lower education with high depressive symptoms reinforces data from previous studies that demonstrated that this variable is a risk factor to the development of mental disorder such as depression [37–40]. Levels of education, as well as the income, are important socioeconomic markers. Studies reveal that the socioeconomic level is associated with several social issues such as low levels of education, unemployment, low-quality housing, and inadequate nutrition. All these factors are directly related to increased levels of depressive symptoms [40]. These conditions may lead to a state of hopelessness and consequently reducing the ability to deal with adversities and frustrating situations.

In addition, our data showed that partners high depressive symptoms were associated with a higher number of severe hypoglycemia episodes as well as the presence of chronic complications in their spouses. Moreover, partners who helped their spouses recovery from severe hypoglycemia had high depressive levels. These findings are in concordance with other studies [1, 21], which highlighted the high level of emotional exhaustion of T1D partners, especially related to hypoglycemia concern and the fear regarding complications.

The high number of serious hypoglycemia episodes were associated with clinically relevant depressive symptoms. It seems that partners exposition to events of serious hypoglycemia of their spouses brings the fear of losing the loved one, by death risk or by serious clinic consequences existent in these episodes [1, 41–44]. The greater number of hypoglycemia episodes, the greater will be the insecurity and exposing to fear. Our study also showed that when partners personally helped their spouses recover from a serious hypoglycemia episode, they had higher levels of depressive symptoms. We observed that besides the partners being exposed to a situation of great anguish, the fact of they must aid a loved one, without the proper training or preparation, it may arouse the feeling of powerlessness and fear. The literature points out that T1D partners reported lack of knowledge and preparation to deal with hypoglycemia. It causes great anguish because they need to help their spouses to recover from hypoglycemia without appropriate support [42, 45]. Plus, the occurrence of serious nocturnal hypoglycemia affects the sleep quality of patients as well as of partners. It might interfere negatively in the day to day functioning, emotional integration, cognitive performance, and alert behavior, bringing a vulnerability and decrease of ability to cope with daily stressors, creating a facilitator scenario to the appearance of depressive symptoms.

It is important to highlight that the most part of T1D partners (73%) of this current study is followed in a tertiary university hospital and there is no diabetes education and emotional support for T1D patients and their family members. The lack of diabetes education and support for T1D family members was demonstrated in DAWN 2 study [19]. Several studies reinforce the need of including T1D partners in educational programs, not only to contribute to disease management of their spouses, but also to emotionally help the family members themselves [43, 45–47].

Therefore, this study emphasizes the need for family members support in the routine of the T1D care and education. Information and psychological support are necessary, not only for patients but also for family members. Our results pointed out some factors associated with high anxiety and relevant depressive symptoms in our partners’ population. Thus, we suggest focusing on these aspects during T1D partners psychological interventions.

Regarding study limitations, the first one is referred to the study design. How this is a cross-sectional study, it does not allow any cause and effect association among the variables studied. Another limitation is related to data collection: sociodemographic and clinic data were self-reported by partners. Moreover, the small number of participants is another study limitation. Because of that, it was not possible to compare patients’ data from public hospital and patients data from private diabetes clinic, which could reveal some particularities due to the socioeconomic differences. Therefore, future researches with a larger number of participants are necessary to confirm the study findings and to elucidate differences between patients from different socioeconomic status. Lastly, another limitation of our study is the lack of information regarding differences between female and male partners.

## Conclusions

This study showed a high frequency of relevant anxiety and depressive symptoms in this T1D partners population.

A higher level of anxiety in partners was associated with the sensor use: CGM.

Lower education and helping their spouses recovery from severe hypoglycemia were associated with high depressive symptoms in T1D partners. Likewise, high depressive symptoms in T1D partners were associated with the following characteristics: higher length of their spouses’ disease, a higher number of chronic complications, and more severe hypoglycemia episodes.

These results emphasize the need to incorporate the psychological and psychiatric aspects into T1D partners education and care. Diabetes education may be a relevant tool to decrease anxiety and depressive symptoms in T1D partners.

## Data Availability

The corresponding author can be contacted for any information related to this study.

## Abbreviations

T1D: Type 1 diabetes
HAD-D: Depression subscale of the Hospital Anxiety and Depression Scale
HbA1C: Glycosylated hemoglobin
HPLC: High-performance liquid chromatography
CGM: Continuous glucose monitoring

## References

[1] Trief PM, Sandberg JG, Dimmock JA, Forken PJ, Weinstock RS (2013). Personal and relationship challenges of adults with type 1 diabetes: a qualitative focus group study. Diabetes Care.

[2] Fisher L, Chesla CA, Skaff MM, Mullan JT, Kanter RA (2002). Depression and anxiety among partners of European-American and Latino patients with type 2 diabetes. Diabetes Care.

[3] Trief PM, Xing D, Foster NC, Maahs DM, Kittelsrud JM, Olson BA, Young LA, Peters AL, Bergenstal RM, Miller KM, Beck RW, Weinstock RS, T1D Exchange Clinic Network (2014). Depression in adults in the T1D exchange clinic registry. Diabetes Care..

[4] Berge LI, Riise T, Hundal O, Odegaard KJ, Dilsaver S, Lund A (2013). Prevalence and characteristics of depressive disorders in type 1 diabetes. BMC Res Notes..

[5] Barnard KD, Skinner TC, Peveler R (2006). The prevalence of co-morbid depression in adults with type 1 diabetes: systematic literature review. Diabet Med.

[6] Fisher L, Hessler DM, Polonsky WH, Mullan J (2012). When is diabetes distress clinically meaningful? Establishing cut points for the diabetes distress scale. Diabetes Care.

[7] Polonsky WH, Fisher L, Hessler D, Johnson N (2016). Emotional distress in the partners of type 1 diabetes adults: worries about hypoglycemia and other key concerns. Diabetes Technol Ther..

[8] Silveira MSVM, Moura Neto A, Sposito AC, Siminerio L, Pavin EJ (2019). Low empowerment and diabetes regimen distress are related to HbA1c in low income type 1 diabetes patients in a Brazilian tertiary public hospital. Diabetol Metab Syndr.

[9] Strandberg RB, Grave M, Wentzel-Larsen T, Peyrot M, Rokne B (2014). Relationships of diabetes-specific emotional distress, depression, anxiety and overall well-being with HbA1c in adult persons with type 1 diabetes. J Psychosom Res.

[10] Snoek FJ, Bremmer MA, Hermanns N (2015). Constructs of depression and distress in diabetes: time for an appraisal. Diabet Med.

[11] Manoogian MM, Harter LM, Denham SA (2010). The storied nature of health legacies in the familial experience of type 2 diabetes. J Fam Commun.

[12] Denham SA, Manoogian MM, Schuster L (2007). Managing family support and dietary routines: type 2 diabetes in rural Appalachian families. Fam Syst Health.

[13] TODAY-Study-Group (2010). Design of a family-based lifestyle intervention for youth with type 2 diabetes: the TODAY study. Int J Obes (Lond)..

[14] Baig AA, Benitez A, Quinn MT, Burnet DL (2015). Family interventions to improve diabetes outcomes for adults. Ann N Y Acad Sci.

[15] Gunn KL, Seers K, Posner N, Coates V (2012). ‘Somebody there to watch over you’: the role of the family in everyday and emergency diabetes care. Health Soc Care Community.

[16] Strom JL, Egede LE (2012). The impact of social support on outcomes in adult patients with type 2 diabetes: a systematic review. Curr Diabetes Rep..

[17] White P, Smith S, O’Dowd T (2009). Understanding type 2 diabetes: including the family member’s perspective. Diabetes Educ..

[18] Beverly E, Miller C, Wray L (2008). Spousal support and food-related behavior change in middle-aged and older adults living with type 2 diabetes. Health Educ Behav..

[19] Kovacs Burns K, Nicolucci A, Holt RIG, Willaing I, Hermanns N, Kalra S (2013). Diabetes attitudes, wishes and needs second study (DAWN2TM): cross-national benchmarking indicators for Family members living with people with diabetes. Diabet Med.

[20] Funnell MM, Bootle S, Stuckey HL (2015). The diabetes attitudes, wishes and needs second study. Clin Diabetes..

[21] Gonder-Frederick L, Cox D, Kovatchev B, Julian D, Clarke W (1997). The psychosocial impact of severe hypoglycemic episodes on spouses of patients with IDDM. Diabetes Care.

[22] Van Dijk M, Donga E, van Dijk JG (2011). Disturbed subjective sleep characteristics in adult patients with long-standing type 1 diabetes mellitus. Diabetologia.

[23] Tracy EL, Berg CA, Baucom KJW (2019). Daily sleep quality and duration and daily stressors in couples coping with type 1. Diabetes Health Psychol.

[24] Zigmond AS, Snaith RP (1983). The hospital anxiety and depression scale. Acta Psychiatr Scand.

[25] Botega Neury J, Bio Márcia R, Zomignani Maria A, Garcia C, Pereira Walter AB (1995). Transtornos do humor em enfermaria de clínica médica e validação de escala de medida (HAD) de ansiedade e depressão. Rev Saúde Pública..

[26] Bjelland I, Dahl AA, Haug TT, Neckelmann D (2002). The validity of the hospital anxiety and depression scale. An updated literature review. J Psychosom Res..

[27] Instituto Brasileiro de Geografia e Estatística (IBGE). 2009. http://www.ibge.gov.br. Accessed 22 July 2019.

[28] European Medicines Agency. Committee for Medical Products for Human Use (CHMP). Guideline on clinical investigation of medical products in the treatment or prevention of diabetes mellitus. Amsterdam: *European Medicines Agency*. 2012. http://www.ema.europa.eu/ema/index.jsp?curl=pages/includes/document/document_detail.jsp?webContentId=WC500129256&mid=WC0b01ac058009a3dc. Accessed 5 July 2013.

[29] American Diabetes Association Workgroup on Hypoglycemia (2005). Defining and reporting hypoglycemia in diabetes. Diabetes Care.

[30] Östenson CG, Geelhoed-Duijvestijn P, Lahtela J, Weitgasser R, Markert Jensen M, Pedersen-Bjergaard U (2014). Self-reported non-severe hypoglycaemic events in Europe. Diabet Med.

[31] Organização Mundial de Saúde-OMS. Depression and other common mental disorders: global health estimates [Internet]. Geneva: WHO; 2017. http://apps.who.int/iris/bitstream/10665/254610/1/WHO-MSD-MER-2017.2-eng.pdf.

[32] Barnard K, James J, Kerr D, Adolfsson P, Runion A, Serbedzija G (2016). Impact of chronic sleep disturbance for people living with T1 diabetes. J Diabetes Sci Technol..

[33] Hiscock H, Wake M (2002). Randomised controlled trial of behavioural infant sleep intervention to improve infant sleep and maternal mood. Br Med J.

[34] Wayte S, McCaughey E, Holley S, Annaz D, Hill CM (2012). Sleep problems in children with cerebral palsy and their relationship with maternal sleep and depression. Acta Paediatr.

[35] Rankin D, Harden J, Waugh N, Noyes K, Barnard KD, Lawton J (2016). Parents’ information and support needs when their child is diagnosed with type 1 diabetes: a qualitative study. Health Expect.

[36] Roberts R, Walsh J, Heinemann L (2014). Help someone is beeping. J Diabetes Sci Technol..

[37] American Psychiatric Association (APA) (2013). Diagnostic and statistical manual of mental disorders—DSM-5.

[38] Boing AF, Melo GR, Boing AC, Moretti-Pires RO, Peres KG, Peres MA (2012). Associação entre depressão e doenças crônicas: um estudo populacional. Rev. Saúde Pública..

[39] Almeida Filho N, Lessa I, Magalhães L, Araújo MJ, Aquino E, James AS, Kawachi I (2004). Social inequality and depressive disorders in Bahia, Brazil: interactions of gender, ethnicity and social class. Soc Sci Med.

[40] Mello MF, Mello AAF, Kohn R (2007). Epidemiologia da saúde mental no Brasil.

[41] Rajaram SS (1997). Experience of hypoglycemia among insulin dependent diabetics and its impact on the family. Sociol Health Illn.

[42] King J, Overland J, Fisher M, White K (2015). Severe hypoglycemia and the role of the significant other: expert, sentry, and protector. Diabetes Educ..

[43] Lawton J, Rankin D, Elliott J, Heller SR, Rogers HA, De Zoysa N (2014). Experiences, views, and support needs of family members of people with hypoglycemia unawareness: interview study. Diabetes Care.

[44] Jørgensen HV, Pedersen-Bjergaard U, Rasmussen ÅK, Borch-Johnsen K (2003). The impact of severe hypoglycemia and impaired awareness of hypoglycemia on relatives of patients with type 1 diabetes. Diabetes Care.

[45] Whittemore R, Delvy R, Mccarthy MM (2018). The experience of partners of adults with type 1 diabetes: an integrative review. Curr Diab Rep.

[46] Orvik E, Ribu L, Johansen OE (2010). Spouses’ educationalneeds and perceptions of health in partners with type 2diabetes. Eur Diabetes Nurs.

[47] Rintala T-M, Paavilainen E, Åstedt-Kurki P (2013). Everyday living with diabetes described by family members of adult people with type 1 diabetes. Int J Fam Med..

